# A Short Executive Function Training Program Improves Preschoolers’ Working Memory

**DOI:** 10.3389/fpsyg.2015.01827

**Published:** 2015-11-24

**Authors:** Emma Blakey, Daniel J. Carroll

**Affiliations:** Department of Psychology, The University of Sheffield, Sheffield, UK

**Keywords:** executive functions, cognitive control, preschoolers, training, working memory, mathematics

## Abstract

Cognitive training has been shown to improve executive functions (EFs) in middle childhood and adulthood. However, fewer studies have targeted the preschool years—a time when EFs undergo rapid development. The present study tested the effects of a short four session EF training program in 54 four-year-olds. The training group significantly improved their working memory from pre-training relative to an active control group. Notably, this effect extended to a task sharing few surface features with the trained tasks, and continued to be apparent 3 months later. In addition, the benefits of training extended to a measure of mathematical reasoning 3 months later, indicating that training EFs during the preschool years has the potential to convey benefits that are both long-lasting and wide-ranging.

## Introduction

Executive functions (EFs) are the set of high level cognitive skills—including working memory, inhibitory control, and cognitive flexibility—that underpin goal-directed behavior (Miyake et al., 2000). Working memory and inhibitory control are two core EFs that develop rapidly during the preschool years, leading to significant improvements in children’s ability to control and regulate their behavior (see Garon et al., 2008). Working memory allows children to maintain and process information, and inhibitory control allows children to suppress distracting information or automatic but task-inappropriate behaviors. During the preschool years these two EFs play an important role in supporting school readiness and children’s developing mathematical skills (Bull et al., 2008; Clark et al., 2010; Best et al., 2011). Understandably, therefore, the question of whether EFs can be improved via cognitive training has received much attention. Surprisingly, however, little of this research has focused on the preschool years—when EFs undergo significant developments.

Despite the importance of the preschool period for EFs (e.g., Zelazo, 2006; Wiebe et al., 2011), very little training research has been conducted with this age group (see Wass et al., 2012 for a review). Many studies have focused on school-age children, and have targeted working memory specifically for training. These studies have shown that working memory training improves children’s performance on non-trained measures of working memory (Jaeggi et al., 2008; Henry et al., 2014; Rode et al., 2014; Karbach et al., 2014). Other studies have targeted multiple EFs during training, including working memory, inhibitory control and planning, and have found that training improves children’s performance on a variety of non-trained measures of EF (Goldin et al., 2014; Traverso et al., 2015). For example, Goldin et al. (2014) trained 6- to 7-year-olds on a 20- to 25-session combined EF training program targeting inhibitory control, working memory and planning. The trained children significantly improved their performance on different non-trained measures of attention, cognitive flexibility, language and mathematics compared to an active control group.

However, despite the fact that there has been a growing interest in classroom and meta-cognitive-based programs aimed at improving self-regulation in preschoolers (see Diamond and Lee, 2011 and Moriguchi et al., 2015), very little computerized EF training research has focused on the preschool years (though see Rueda et al., 2005a; Thorell et al., 2008; Bergman Nutley et al., 2011). This is a surprising oversight: a strong argument has been made that interventions may *a priori* be more successful in younger children due to greater plasticity in relevant neural networks (Wass, 2015). Consistent with this view, transfer from EF training is greater in younger children than older children (Melby-Lervåg and Hulme, 2013), with significant negative correlations being found between the age of the participants and the transfer observed (Wass et al., 2012). Thus, a renewed focus on cognitive training in the preschool period is clearly needed.

We highlight two questions of particular interest addressed by this study. The first is the extent to which cognitive training improves EF, as opposed to merely improving performance on a specific task. Progress in answering this question has been hindered by the use of transfer tasks that share the same surface features as the trained tasks, as well as by the use of passive control groups (Shipstead et al., 2012; Green et al., 2014). The second question regards possible benefits of training gains to mathematical skills. This is important, since much of the utility of EF training rests on its effectiveness in improving real-life outcomes. Of the few studies that have looked at this, three found no transfer to mathematical skills (Dunning et al., 2013; Henry et al., 2014; Karbach et al., 2014), and two did find transfer to mathematical skills (Goldin et al., 2014; Rode et al., 2014). This question remains unaddressed in preschoolers. Furthermore, based on current research, it is unclear how intensive training interventions need to be in order to produce a lasting effect. Training improvements have been reported after as many as 25 sessions and as few as three sessions (see Karbach and Unger, 2014 and Wass, 2015 for a review). The potential impact of cognitive training is likely to be inversely proportional to the time and effort required to bring about improvements. The effectiveness of shorter training programs is therefore of particular interest.

The present study examined whether a short EF training program improved non-trained EFs in preschoolers, using a randomized-control pre-test post-test design. Importantly we use a short four-session program delivered over 1 month, and target working memory and inhibitory control, two core EFs in early childhood (Garon et al., 2008; Hughes et al., 2009; Chevalier et al., 2012) thought to be critical for children’s developing mathematical ability (Raghubar et al., 2010; Gilmore et al., 2013). The study had three key aims: first, to examine whether the benefits of training transfer to working memory and inhibitory control tasks that share very few surface features with the trained tasks, and also to assess far transfer to cognitive skills not targeted in the training program (namely cognitive flexibility and processing speed). Second, to examine whether transfer effects are maintained 3 months post-training. Third, as preschool EF has been consistently shown to relate to mathematical ability, but standardized scores of mathematical ability are not available for UK children under 5 years (Wechsler, 2005), measures of mathematical ability were taken at the 3-month follow-up once children had started school to examine whether the training program leads to benefits in children’s mathematical skills.

## Materials and Methods

### Design

Children completed baseline measures of working memory, inhibitory control, cognitive flexibility and processing speed. They were then randomly assigned to either the Training group or the Control group, with the sole constraint that children from each of the two participating preschools were distributed equally across the two conditions. Both groups completed 4 weekly 20-min sessions of computerized tasks. Baseline measures were readministered 1 week after training (the post-test session), and again 3 months later (the follow-up session). Measures of mathematical ability were included at the 3-month follow-up.

### Participants

Initially, 60 children were recruited from two preschools in lower to middle-class areas of the UK. Five children missed a training session, and one did not understand the task instructions, so the final sample comprised 54 children: 26 in the Training group (*M* age = 4;4 years, SD = 3.65; 13 males, 13 females), and 28 in the Control group (*M* age = 4;4 years, SD = 3.50; 14 males, 14 females). Informed consent was obtained from teachers and caregivers. Children received a small gift after the final session. Ethical approval was obtained from the University’s Psychology ethics sub-committee.

### Procedure and Materials

Children were tested individually in their preschool. All training and control tasks were administered on an Iiyama touchscreen connected to a PC running E-Prime software (Psychology Software Tools, Pittsburgh, PA, USA). To reduce incidental between-group differences, identical stimuli were used in the training and control tasks, and feedback was provided in all tasks. To maintain interest in the tasks, stimuli varied between each session (for example, from jungle animals to farmyard animals). To check for baseline differences in classroom engagement, one teacher in each preschool blind to children’s condition rated children using the teacher-rated Classroom Engagement Scale (Pagani et al., 2010). In addition, to check for differences in motivation between groups, at the end of the 4 weeks all children indicated on an age-appropriate Likert scale how much they had enjoyed playing the games (from 1, “A lot,” to 4, “Not at all”).

### Training Tasks

Four training tasks were used adapted from existing established measures of preschool EF. Two targeted aspects of working memory: the Six Boxes task (Diamond et al., 1997) and the One-back task (Tsujimoto et al., 2007); two targeted aspects of inhibitory control: interference control (the Flanker task, Rueda et al., 2005b) and response inhibition (the Go/No-Go task, Simpson and Riggs, 2006). Each task lasted approximately 5 min. As preschoolers’ performance is known to be variable within EF training tasks (van Bers et al., 2011), we sought to minimize within-task variance by using an adaptive training regime between each session such that if children were accurate on 75% or more of trials in a session, the level of difficulty for a particular task increased in the following session. Tasks were administered in the same order: the Six Boxes task, followed by the Flanker task, the One-back task, and the Go/No-Go task.

### Working Memory Training

Six Boxes task: children were asked to find rewards (e.g., stickers) hidden behind six different objects (e.g., colored boxes). To begin with, all of the objects hid a reward. If children selected the correct object, the reward would be revealed. If they were incorrect (i.e., by selecting an object they had already searched), no reward was revealed. Therefore, children could only find reward behind objects not already searched. Between trials, objects were moved so that their locations were different to the previous trial. Children completed this task twice consecutively in each session. The game ended when children had found all of the rewards. The dependent variable was the number of trials taken to find all items across both tasks. In the first training session, the ISI lasted for 4000 ms. If children scored 75% correct or better, the duration of the ISI was increased by 2000 ms (to a maximum of 8000 ms).

One-back task: children were shown a succession of images (e.g., animals), presented one at a time. Children were told to touch the image on the screen if it matched the image that had appeared on the preceding trial. Children completed three blocks of 20 test trials (of which one-third were “hit trials,” whereby the image shown had also appeared on the previous trial, and therefore required a response). The dependent variable was hit-trial accuracy. Images were presented for 2000 ms with an ISI of 1000 ms. If children were accurate on 75% or more of trials, the ISI in the next session increased by 1000 ms (to a maximum of 3000 ms).

### Inhibitory Control Training

Flanker task: children were presented with a line of 5 stimuli (e.g., rockets) and asked to indicate which direction the central stimulus was facing (left or right). Children completed three blocks of 20 test trials. Half the trials were congruent (stimuli were all left-facing or all right-facing); and half were incongruent (the middle stimulus faced the opposite direction to the flanking stimuli). Stimuli were presented for 4000 ms, with a fixation in between trials lasting 1000 ms. If children were accurate on 75% or more trials in a session, the amount of time that stimuli appeared on the screen was reduced by 1000 ms (to a minimum of 2000 ms).

Go/No-Go task: children were required to touch a series of stimuli appearing on the screen (e.g., a fish) but to make no response when a specific “no-go” stimulus appeared (e.g., a shark). Children completed three blocks of 20 test trials (Go:No-go trial ratio 2:1). In the first session, the stimuli appeared on screen for 2000 ms. If children were accurate on 75% more of no-go trials, this time reduced to 1500 ms, and again to 1200 ms.

### Active Control Tasks

The Control group completed three tasks that required children to make simple perceptual judgements. The first task required children to decide whether two pictures were the same or different; the second task required children to search for a particular image amongst distractors (for example, “find the cat in the tree”); and the third task required children to decide which of two pictures had more objects in it. The control tasks used the same stimuli and lasted the same duration as the training tasks.

### Baseline Measures

To assess training improvements, five tasks were administered at three different time points: 1 week prior to training (baseline), 1 week after training (post-training), and 3 months post-training (follow-up). EF tasks were chosen specifically because they did not share the same surface features or instructions as the training tasks. Tasks were administered in the following fixed order: the SwIFT, the Backward Word Span, the Peg-tapping task, the FIST, and the Bubble-popping task. In addition, two tasks measuring mathematical ability were administered at the 3-month follow-up.

### Working Memory

In the Backward Word Span (Davis and Pratt, 1996), children were shown pictures of familiar objects one at a time (e.g., a cat and a spoon) and were asked to recall them in a backward order. Children completed two practice trials and then up to nine experimental trials, three of each span length (two, three and four). If children got at least two out of the three trials correct, the span length increased. The dependent variable was the number of trials correctly recalled in a backward order.

### Inhibitory Control

In the Peg-tapping task (Diamond and Taylor, 1996), children were instructed to tap twice with a stick when the experimenter tapped once; and to tap once when the experimenter tapped twice. After watching a demonstration from the experimenter, children completed twelve trials in a fixed pseudo-random order (six of each rule, with no more than three consecutive trials of one rule). The dependent variable was the correct number of responses.

### Cognitive Flexibility

The SwIFT (Switching, Flexibility and Inhibition Task: Blakey et al., in press) was a rule-switching task administered using a touchscreen computer. Children had to match colorful shapes on the relevant dimension for that trial (either color or shape). Children completed a pre-switch phase of eight trials using one rule; then a post-switch phase of eight trials using a different rule; and finally two mixed blocks of 12 trials each where the rule switched in a pseudo-random order between trials (no more than four repetitions of a rule). Each trial began with a prompt stimulus appearing at the top of the screen. After a delay of 1000 ms, two response stimuli appeared in the lower left and right corners of the screen. One stimulus was the target (the correct response, as it matched the prompt on the currently relevant dimension), and the other was a distractor (the incorrect response). The distractor always matched the prompt on the non-relevant dimension. The distractor and target were equally likely to appear on the lower left or right corner of the screen. Children were given rule reminders on every trials using a pre-recorded instruction (e.g., “touch the one that’s the same color”). Rule order was counterbalanced. The dependent variables were post-switch accuracy and mixed-block accuracy.

In the FIST (flexible item selection task; Jacques and Zelazo, 2001), children were presented with three different pictures of familiar objects and asked to select two pictures (out of three) that go together on one dimension (such as color, shape or size), and then to select two pictures that go together on a different dimension. For example, on one trial children were presented with a small pink flower, a small green ball and a large blue ball. Therefore children could select the small flower and small ball because they match on size, and the green ball and the blue ball because they match on shape. There were twelve trials. The dependent variable was a proportion score, calculated by dividing the number of correct responses for selection two by the total number of correct trials for selection one.

### Processing Speed

Processing speed was measured by a simple task in which children “popped” bubble stimuli appearing on a touchscreen computer by touching them as quickly as they could. When children touched the stimulus, a picture of a burst bubble appeared in its place. Children were given a short demonstration. There were then eight test trials. The ISI varied randomly between 800 and 1200 ms. The dependent variable was the mean reaction time.

### Mathematical Ability

Two measures of mathematical ability from the Wechsler Individual Achievement Test-II battery were used: Numerical Operations and Mathematical Reasoning (Wechsler, 2005). The Numerical Operations subtest comprised 22 questions assessing children’s ability to identify, write, and count numbers, and to solve arithmetic calculations. The Mathematical Reasoning subtest comprised 30 questions assessing children’s ability to identify shapes, extract information, and solve multi-step word problems. Standardized scores for each task were used as the dependent variable.

## Results

Means and standard deviations for both groups are reported in Table 1. Correlations between the tasks at baseline are reported in Table 2. For significant transfer effects, in addition to partial eta square, Cohen’s *d* is reported—or the standardized mean difference in performance between pre and post-test for each group. First, we checked for baseline differences between the groups. There were no group differences in age, self-rated enjoyment, teacher-rated classroom engagement, or on any of the tasks at baseline (all *p*s > 0.10).

**TABLE 1:** **Mean scores by group at the pre, post, and 3 month follow-up assessments**.

****	**Active Control Group**			**Training group**		
	**Pre (T1)**	**Post (T2)**	**Follow-up (T3)**	**Pre (T1)**	**Post (T2)**	**Follow-up (T3)**
Backward word	2.43 (1.50)	2.75 (1.62)	3.32 (2.04)	2.54 (1.82)	3.69 (1.93)	4.96 (1.64)
Peg tapping	7.43 (4.56)	8.79 (3.78)	10.20 (3.12)	7.73 (3.66)	10.85 (1.49)	11.17 (1.44)
Post-switch	6.46 (2.24)	6.46 (2.35)	7.32 (1.03)	6.38 (2.58)	6.81 (1.96)	7.00 (2.27)
Mixed switch	15.64 (4.36)	17.61 (4.72)	19.60 (3.64)	16.88 (4.69)	18.19 (3.80)	20.17 (3.64)
FIST	0.72 (0.22)	0.78 (0.21)	0.83 (0.16)	0.73 (0.24)	0.80 (0.20)	0.84 (0.20)
Proc speed (ms)	1114 (281)	1105 (457)	975 (264)	1089 (295)	925 (200)	931 (195)
Classroom eng	2.51 (0.36)			2.61 (0.43)		
Num ops			89.67 (8.69)			92.91 (11.54)
Maths reasoning			98.83 (8.62)			103.64 (6.45)

Standard deviations are in parentheses and mathematical scores are standardized.

**TABLE 2:** **Correlations between the tasks at baseline**.

****	**1**	**2**	**3**	**4**	**5**	**6**	**7**	**8**	**9**
1. Backward word		0.24^†^	0.01	0.27*	0.25^†^	–0.05	–0.00	0.31*	0.33*
2. Peg tapping			–0.01	0.32*	0.28*	–0.10	0.37**	0.45**	0.53**
3. Post-switch				0.26^†^	0.12	0.07	0.19	0.12	0.22
4. Mixed switch					0.47**	0.27*	0.30*	0.18	0.27^†^
5. FIST						0.01	0.27*	0.23	0.14
6. Proc speed							–0.32*	–0.14	–0.26^†^
7. Classroom eng								0.08	0.16
8. Num operations									0.61**
9. Maths reasoning									

N = 59, ^†^p < 0.10, *p < 0.05, **p < 0.01. Note that the Numerical Operations and Mathematical Reasoning sub-tests were included at the 3 month follow-up.

Each training task had three levels of difficulty; children progressed to a higher level of difficulty as a function of good performance on a previous level. Because task accuracy on different levels of difficulty are thus not directly comparable, we only examine these data descriptively. For the Six Boxes task, 62% of children improved over training, of whom 75% reached the highest level by the final session. For the One-back task, 96% of children improved over training, with all of these reaching the highest level by the final session. For the Flanker task, 73% of children improved over training, of whom 74% reached the highest level by the final session. For the Go/No-Go task, all children improved over training, with 92% reaching the highest level by the final session.

To test whether training transferred to the five non-trained EF tasks, general linear models were performed separately for each task with time (baseline vs. post-test) and group (Training vs. Control) on each task score. For working memory, there was a significant main effect of time, [*F*(1,52) = 13.21, *p* = 0.001, η^2^_*partial*_ = 0.20] and no significant effect of group, [*F*(1,52) = 1.55, *p* > 0.1]. Importantly, this was qualified by a significant interaction between time and group, [*F*(1,52) = 4.21, *p* = 0.045, η^2^_*partial*_ = 0.08]. Only children in the Training group significantly improved from baseline on the Backward Word Span (*d* = 0.61) and not the active control group (*d* = 0.20) (see Table 1).

There were significant main effects of time on: inhibitory control; FIST performance; and mixed block accuracy on the SwIFT. This indicates that children in both groups got more accurate on these tasks from pre-test to post-test (all *p*s < 0.05, η^2^_*partial*_ = 0.08 –0.22). There were no significant main effects of time on post-switch accuracy on the SwIFT (*p* > 0.1), nor on processing speed (*p* > 0.1), indicating that children did not get better on these tasks from pre- to post-test. There was no significant main effect of group on: inhibitory control; FIST performance; SwIFT post-switch accuracy and mixed block accuracy; nor processing speed (all *p*s > 0.1). Finally, there were no other significant interactions between time and group on any other task (all *p*s > 0.05).

To test whether the effect of training on working memory persisted at the 3-month follow up, a general linear model was performed as described above. Six children were unavailable for testing at the 3-month follow-up, leaving a final sample of 48 children (Training group *N* = 23; Control group *N* = 25). There was a significant main effect of time on working memory, [*F*(1,46) = 37.66, *p* < 0.001, η^2^_*partial*_ = 0.45] and no significant effect of group, [*F*(1,46) = 3.61, *p* > 0.05]. This was qualified by a significant interaction between time and group, [*F*(1,46) = 9.18, *p* = 0.004, η^2^_*partial*_ = 0.17]. Only the training group (*d* = 1.40) improved from baseline on the Backward Word Span at the 3-month follow-up and not the active control group (*d* = 0.50) (see Table 1).

Two additional tasks were included at follow-up: Mathematical Reasoning and Numerical Operations. A further two children were unavailable for this testing session, leaving a final sample of 46 children. A one-way ANOVA found an effect of training on Mathematical Reasoning, *F*(1,44) = 4.51, *p* = 0.039, η^2^_*partial*_ = 0.09, *d* = 0.63. Children in the Training group scored higher on Mathematical Reasoning than children in the Control group (see Table 1). The effect remained when baseline working memory was included as a covariate, *F*(1,43) = 4.71, *p* = 0.036, η^2^_partial_ = 0.10. There was no effect of training on the Numerical Operations sub-test, *F*(1,44) = 1.17, *p* = 0.29.

To examine whether working memory improvements were related to training gains, Spearman’s rank-order correlations were run between the level obtained in the final session of training (lowest, intermediate, or highest level) on the Flanker and the Six Boxes task only (since performance reached ceiling on the One-back and Go/No-Go tasks) and a mean difference score calculating improvement on the Backward Word Span between pre and post-test. For the Six Boxes task, 10 children stayed at the lowest level, four children got to the intermediate level and twelve children reached the highest level. There was a significant positive correlation between improvements in working memory and training gains on the Six-Boxes task, *r*_*s*_(26) = 0.47, *p* = 0.02. For the Flanker task, seven children stayed at the lowest level, five children got to the intermediate level and 14 children reached the highest level. There was a significant positive correlation between improvements in working memory and training gains on the Flanker task, *r*_*s*_(26) = 0.60, *p* = 0.001. Working memory improvements at the 3-month follow-up were not significantly correlated with training gains on either the Six Boxes task or the Flanker task (*p* > 0.1). Mathematical Reasoning performance was also significantly positively correlated with both training gains on the Six Boxes task [*r*_*s*_(22) = 0.53, *p* = 0.01] and the Flanker task [*r*_*s*_(22) = 0.58, *p* = 0.01].

## Discussion

A four-session EF training program significantly improved preschoolers’ working memory. Specifically, the improvement was found on the Backward Word Span, a task sharing few surface features with—but the same underlying EF demands as—the training tasks. Importantly, this indicates genuine training of EFs, and not simply increased familiarity with a particular task. This point is further emphasized by the fact that improvement on an untrained working memory task was significantly associated with the degree of improvements on the trained measures of working memory and inhibitory control. Furthermore, the benefit of training on working memory was maintained 3 months post-training. This is an important finding, as if training is to have genuine utility as a basis for future interventions, it is essential that its effects are not merely transient, but persist over time. This enduring effect in the current study is striking, given the relatively short training program involved. These results with preschoolers are consistent with previous research demonstrating the maintenance of cognitive training benefits in older children (e.g., Dunning et al., 2013), and provide new evidence that shorter programs may be as effective as longer programs. This is particularly important given the positive impact this could have on children before they start school and begin learning in a structured environment—something children with poor working memory find particularly difficult (Gathercole et al., 2008).

The training program had unique benefits for preschoolers’ working memory. No evidence of transfer to inhibitory control or far transfer to cognitive flexibility was found—a pattern of results consistent with other studies, which taken together suggest that working memory may be particularly amenable to training (Thorell et al., 2008; Karbach et al., 2014). Typically, improvements in cognitive flexibility only occur following computerized training programs specifically targeting cognitive flexibility (e.g., Karbach and Kray, 2009; Zinke et al., 2012). This may be due to the complexity of cognitive flexibility: it is generally considered to be an emergent EF arising from multiple cognitive skills including attention, meta-cognition, working memory and inhibitory control (Garon et al., 2008; Blakey et al., in press). It is possible that our training program was not intense enough, or did not tap enough cognitive skills, to improve cognitive flexibility. Likewise, we did not find transfer to inhibitory control despite improvements in inhibitory control over training (a result also found by Thorell et al., 2008). However, improvements in inhibitory control and working memory over training *did* transfer to a non-trained measure of working memory. One possible explanation for this is that our EF training program was more successful at training interference control than response inhibition and therefore was more likely to transfer to a non-trained measure of working memory than response inhibition. These different facets of inhibition are considered distinct processes at both the behavioral and the neural level (Nigg, 2000; Verbruggen et al., 2004; Martin-Rhee and Bialystok, 2008; Groom and Cragg, 2015), and interference control is known to be important for working memory (Kane and Engle, 2003; Redick and Engle, 2006).

Children in the Training group showed better Mathematical Reasoning compared to the control group 3 months post-training. This is a finding of potentially great importance. To our knowledge, only two studies have found evidence that EF training improves mathematical ability in children (Goldin et al., 2014; Rode et al., 2014). It is interesting to note that of the two studies that find this effect, Goldin et al. (2014) targeted a variety of EFs known to be important for mathematical skills (as in the present study), and Rode et al. (2014) incorporated mathematical problems within the training tasks. This may suggest that for domain-general training programs, it is important to target both inhibitory control and working memory in order to see benefits to children’s mathematical skills. Given prior research on the importance of both working memory and inhibitory control to children’s developing mathematical skills, it is plausible that training these EFs might lead to benefits for mathematical skills. However, it is remarkable that in the present study we see benefits 3 months post-training and after such a short training program. It is notable that the effect was specific to Mathematical Reasoning. The reasoning sub-test requires children to engage in multi-step operations, whereas Numerical Operations focus on retrieval of information from long-term memory, with the former thought to place more demands on working memory (Titz and Karbach, 2014).

Because measures of mathematical skill were only available at follow-up, we cannot rule out the possibility that this difference arose due to undetected between-group differences in mathematical ability. However, we think this unlikely: the two samples were very similar in cognitive ability, as there were no baseline differences between groups in terms of age, processing speed, classroom engagement or EF; and the effect of training on Mathematical Reasoning remained even when baseline working memory was included as a covariate. However, based on the current study design, we cannot definitively rule out this possibility. A further limitation of the current study was that in assessing transfer to academic skills, we only included measures of mathematical skills. In addition to mathematical skills, EFs have been linked to children’s literacy and self-regulation in the classroom (see Blair and Razza, 2007). Therefore, future cognitive training research would benefit from including a wider variety of academic achievement measures to examine how far cognitive training can enhance a range of real-life outcomes for children.

While the question of how far cognitive training transfers to academic skills remains open, the present study shows that such training has the potential to support and improve EFs during childhood. A relatively short training program of just four sessions led to a specific improvement in working memory that was maintained after a 3 months. This raises an interesting suggestion for future research that focusing training interventions on preschoolers may be one way to scaffold cognitive development before children start school.

## Author Contributions

EB designed the study and DJC advised on the design. EB collected and analysed the data. EB wrote the manuscript and DJC provided critical revisions. Both EB and DJC have approved the final version of the manuscript and agree to be accountable for all aspects of the work.

### Conflict of Interest Statement

The authors declare that the research was conducted in the absence of any commercial or financial relationships that could be construed as a potential conflict of interest.
